# Refractory Nausea and Vomiting Due to Central Nervous System Injury: A Focused Review

**DOI:** 10.3390/life15071021

**Published:** 2025-06-27

**Authors:** Stefan Stoica, Christopher Hogge, Brett James Theeler

**Affiliations:** 1 F. Edward Hébert School of Medicine, Uniformed Services University of the Health Sciences, Bethesda, MD 20814, USA; stefan.stoica@usuhs.edu; 2 Department of Neurology, Walter Reed National Military Medical Center (WRNMMC), Bethesda, MD 20814, USA; christopher.j.hogge.mil@health.mil; 3 Department of Neurology, F. Edward Hébert School of Medicine, Uniformed Services University of the Health Sciences, Bethesda, MD 20814, USA

**Keywords:** nausea, vomiting, area postrema syndrome, circumventricular organ, chemotherapy, nucleus tractus solitarius, neuromyelitis optica

## Abstract

The area postrema (AP) is a circumventricular organ (CVO) at the base of the fourth ventricle. It has a crucial role in regulating nausea and vomiting due to its unique blood–brain barrier (BBB)-permeability and extensive neural connectivity. Here, we present two cases of area postrema syndrome (APS), a rare condition of intractable nausea and vomiting resulting from direct AP injury. Our cases each occurred in the context of infratentorial neoplasms or their treatment. Using these cases as a framework, we review the literature on central emetic pathways and propose a treatment algorithm for managing refractory nausea and vomiting of central origin. We also highlight other targets beyond conventional serotonergic, dopaminergic, or histaminergic blockade and their roles in central hyperemesis. Our literature review suggests that APS is due to the disruption of the baseline inhibitory tone of outgoing AP signals. When other options fail, our algorithm culminates in the off-label use of combined serotonergic and neurokinin-1 blockade, which is otherwise used to manage chemotherapy-induced nausea and vomiting (CINV). We believe multimodal CNS receptor blockade is efficacious in APS because it addresses the underlying central neural dysregulation, rather than solely targeting peripheral emetic triggers.

## 1. Introduction

### 1.1. Background

The area postrema (AP) is a circumventricular organ (CVO) found in the rostral medulla, at the base of the fourth ventricle. It functions as a potent regulator of nausea and vomiting [1,2]. The emetic reflex is controlled by the dorsal vagal complex (DVC), which is primarily composed of the AP and the nucleus tractus solitarius (NTS) [3]. The DVC receives inputs from a variety of modalities, including enteric vagal afferents, molecules in circulation, brainstem vestibular nuclei, and the insular cortex [3,4,5,6,7,8]. After collecting information derived from these internal stimuli, the emetic response culminates in an efferent signal executed by the dorsal motor nucleus of the vagus (DMX). While emesis is an observable behavior in response to actual stimuli (e.g., bacteria, cardiac ischemia, and toxins) or perceived stimuli (e.g., the emotion of “disgust”), nausea is the abstract sensation of internal sickness that often, but not necessarily, progresses towards emesis [3].

Intractable nausea and vomiting are major therapeutic challenges due to their effects on patient safety and satisfaction. Chronic nutritional losses can manifest with unintended weight loss and vitamin deficiencies. Simultaneously, the psychological stress of avoiding occupational or social settings puts strain on the patient’s support systems. Due to the various mechanisms in which intractable nausea and vomiting can occur, clinicians may need to agonize or antagonize multiple receptors in order to achieve long-lasting relief for patients [9,10].

Clinically, modulating the AP’s function is most frequently performed in the management of chemotherapy-induced nausea and vomiting (CINV) [2,11,12]. Stimulation of intestinal enterochromaffin cells by orally ingested chemotherapeutics causes local release of serotonin, which then binds to enteric vagal afferents to send a corresponding signal to the AP and NTS. Simultaneously, direct chemosensation of circulating toxins causes substance P (SP)-mediated initiation of the emetic response [11]. As a result, some of the most potent antiemetic agents are targeted inhibitors of the type-3 serotonin receptor (5HT-3) and neurokinin-1 (NK-1) SP receptor, both of which are robustly expressed in the AP [13]. Despite the key role of serotonergic signaling in the emetic pathway, there are a litany of other receptors and neurocircuitry that serve as therapeutic targets to treat nausea and vomiting. Dopamine type 2 (D2) receptors, calcitonin gene-related peptide (CGRP) receptors, the endocannabinoid system, and histaminergic (H1), muscarinic, and gastric peptide hormone receptors are all widely distributed in the dorsal vagal complex [14,15,16]. These receptors, especially D2, H1, and the muscarinic types, are common targets of antiemetics.

While CINV is an example of a physiological response to exogenous compounds, a syndrome of intractable nausea and vomiting can also occur in the setting of direct damage to central nervous system (CNS) emetic centers like the AP. These scenarios pose a significant clinical challenge because, unlike with peripheral triggers, when CNS circuitry becomes compromised there may not be a predictable response to the first- or second-line antiemetic regimens. We describe two cases of refractory nausea and vomiting that occurred after treatment of infratentorial neoplasms, and which were eventually successfully treated with off-label use of netupitant and palonosetron [17]. We review the literature describing therapeutic molecular targets of emesis, and suggest a treatment algorithm for refractory, lesional nausea and vomiting.

### 1.2. Area Postrema Syndrome

The presentation of undifferentiated nausea and vomiting has a broad differential diagnosis. Clinicians must take careful note of the patient’s history, medical conditions, medications, substance use, toxic exposures, and physical exam findings to best determine a peripheral or central source for their symptoms [18]. Most cases of vomiting are treated with supportive measures, which may include a brief course of antiemetics.

On the other hand, area postrema syndrome (APS) is a consequence of lesional damage to the AP, typically confirmed with neuroimaging, that can produce some combination of intractable nausea, vomiting, or hiccups [19]. Because of its location in the CNS, APS often coincides with other neurological deficits, depending on whether neighboring structures in the brainstem or cerebellum were also affected by the injury. Unlike most causes of vomiting, APS is resistant to first-line antiemetics, and is usually managed by identifying and treating its underlying cause.

The most common cause of brainstem-localized hyperemesis is its incidence as secondary to a neuromyelitis optica spectrum disorder (NMOSD), an autoimmune demyelinating disorder caused by autoantibodies to Aquaporin 4 (AQP4) [20,21]. AQP-4 is a protein widely expressed on ependymal cells and astrocyte endfeet processes surrounding CVOs, including the AP. Resolution of APS caused by NMOSD is managed by treating the autoimmune, inflammatory response with intravenous immunoglobulins, plasma exchange, glucocorticoids, and monoclonal antibodies [20,21]. However, cases of APS caused by a mass impinging on the AP or through ischemia of the AP are rare and not well-described in the literature. Unlike in NMOSD, the targeted damage to the DVC with infarct- or mass-lesions is not readily reversible because prescribing anti-inflammatories fails to address the mechanism of injury.

Central regulation of nausea and vomiting involves a complex interplay among multiple brainstem nuclei responding to ascending endogenous signals and descending cortical inputs, and ascribing emotional valence to somatic sensations. There is limited number of available works in the literature on the treatment of the central causes of intractable emesis, and clinicians are faced with challenging decisions when presented with patients whose nausea and vomiting respond poorly to first-line medications. Much of the current treatment for any cause of vomiting is extrapolated from chemotherapy-related indications, which may have key differences with respect to centrally induced emesis. The AP exerts an overall inhibitory tone on the sensation of nausea and motor reflex of vomiting, which can be overcome by ascending gastrointestinal vagal inputs or direct chemosensation of chemicals in the serum [15,22]. When the AP is directly injured, it is possible that the damage produces a hyperemetic state. Failure to adequately respond to conventional antiemetics could be due to inadequate blockade of the excitatory central emetic circuits driven by substance P or serotonin. Likewise, a poor response to first-line antiemetics may also be due to medications that act primarily on enteric receptors, and thus do not address the CNS localization of the pathologic emetic response.

## 2. Case Examples

### 2.1. Patient 1

**Figure 1:**
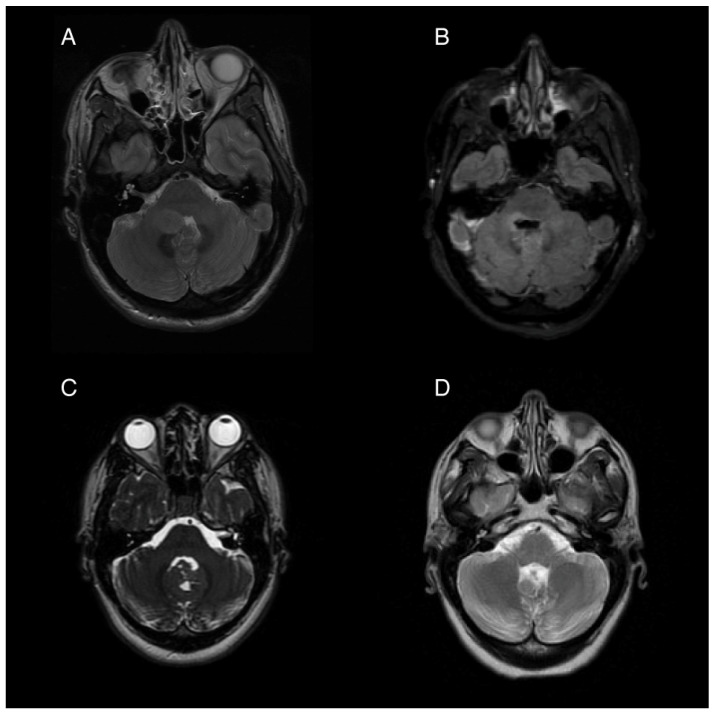
Pre- and post-operative magnetic resonance imaging (MRI) of two patients with posterior fossa neoplasms. (**A**) Pre-operative T2 sequence of Patient 1, showing a 1.9 × 1.5 cm ovoid mass in the right cerebellar hemisphere extending into the fourth ventricle. (**B**) Post-operative T2 FLAIR image showing postsurgical changes with pontine and medullary hyperintensity. (**C**) Pre-operative T2 from Patient 2 showing an iso-intense mass partially obstructing the fourth ventricle and extending from the cerebellar vermis. (**D**) Post-operative T2 showing postsurgical volume loss of the inferior vermis.

A 35-year-old woman presented to the hospital with a 2-day history of nausea, vomiting, and headaches. Neurological examination showed diffuse hyperreflexia and subtle right-sided dysdiadochokinesia. Imaging revealed an ovoid, non-enhancing expansile lesion in the right cerebellar hemisphere measuring 1.9 × 1.5 cm, affecting the right middle cerebellar peduncle and extending through the fourth ventricle (Figure 1A). Stereotactic biopsy followed by a gross total resection was performed, and the histology was consistent with pilocytic astrocytoma. An activating PTCH1 mutation was found in next generation sequencing, with the medulloblastoma tumor type confirmed by DNA methylation profiling.

Following tumor resection, the patient developed an ataxic gait, right upper extremity dysmetria, an extraocular “one and a half syndrome,” nausea, and vomiting. For two months post-operatively, the nausea was controlled with ondansetron, 4 mg three times daily, and promethazine, 12.5 mg every 6 h as needed for breakthrough events. Four months post-operatively, she started to experience intractable nausea and vomiting unresponsive to ondansetron and promethazine. She experienced frequent episodes of vomiting without provocation, as well as nausea and vertigo that sometimes, but not always preceded the vomiting. Her ability to participate in rehabilitation was severely limited by her intractable and unpredictable episodes of moment-to-moment nausea and vomiting. Regimens including oral granisetron, a granisetron patch, and olanzapine in combination provided incomplete relief. A trial of oral aprepitant for 3 days in combination with oral granisetron resulted in a significant improvement for about 1 week, but the symptoms returned. Her refractory vomiting four months after surgery was attributed to localized damage to the area postrema, evidenced by the post-operative edema at the base of the fourth ventricle seen on post-operative imaging (Figure 1B).

In order to introduce a long-acting combination of NK-1 and 5HT-3 antagonism, off-label use of a combination medication comprising netupitant and palonosetron was initiated. This medication is approved to treat chemotherapy-induced nausea and vomiting. The patient experienced full remission of her symptoms within the first week of treatment. Olanzapine was continued at 5 milligrams daily, and over the next 4 months she was able to taper off all of her antiemetic medications. In another seven months, her nausea and vomiting had completely resolved without the use of any daily medications. By 12 months after surgery, she had no need for any as-needed antiemetics.

### 2.2. Patient 2

A 26-year-old woman presented for management following gross-total resection of a medulloblastoma, WNT-activated subtype, WHO grade 4 affecting her cerebellar vermis (Figure 1C). After the surgery the patient began experiencing daily (usually at least twice per day) episodes of sudden vomiting. These episodes were unrelated to meals and occurred without concurrent symptoms of vertigo or nausea. She had considerable anxiety with respect to her vomiting, which contributed to poor nutritional intake and a 14 kg weight loss in the month following her surgery. While the remainder of her neurological exam was mostly normal, she was also experiencing significant fatigue, dyspnea, and a sensation of instability without frank vertigo, requiring her to push a wheelchair for gait assistance.

Her episodic vomiting was attributed to APS caused by partial resection of the inferior cerebellum and an irritation of the dorsal medulla which was shown on imaging (Figure 1D). Notably, she did not have other symptoms of central vestibulopathy, particularly vertigo. Her vomiting was neither related to episodic vertiginous symptoms nor associated with body position or movement. She was initially started on a course of granisetron and aprepitant for a 5-day trial, which resulted in a reduction in her episodes of vomiting, but she then experienced a return of the vomiting episodes after 7 days. After this trial, treatment with weekly netupitant and palonosetron (combination antiemetic capsule) was initiated. She had a near complete resolution of her vomiting while on weekly netupitant and palonosetron.

Two months after resection, she started proton craniospinal irradiation (CSI) and she stopped all antiemetics during radiation treatment. She had a return of her vomiting syndrome a few weeks into the radiation treatment, and at follow-up one-month post-CSI, netupitant and palonosetron once weekly were again restarted. The recurrence of her nausea and vomiting several weeks into CSI may have been due to the effects of radiation. Cranial radiation may contribute to inflammation and necrosis of the AP, similar to how radiation to the abdomen is thought to induce vomiting by direct damage to enterochromaffin cells [23]. At that same one-month follow-up, she began a carboplatin, cyclophosphamide, and etoposide (CCE) adjuvant chemotherapy regimen. On weekly netupitant and palonosetron, she had complete resolution of her vomiting syndrome, allowing her to gain weight and eat normal meals during chemotherapy. The plan at the end of 6 months of chemotherapy was to increase the time between the doses of netupitant and palonosetron to beyond 7 days, with the goal of eventually stopping the medications.

## 3. Central Nervous System Targets of the Emetic Response

### 3.1. Inflammatory Cytokines

Mechanistically, the dorsal vagal complex contains a multitude of immune receptors including interleukin 6 (IL-6) and toll-like receptor 4 (TLR-4), and prostaglandin receptors which are partially responsible for the nausea and cachexia associated with systemic inflammation [3,24,25]. Furthermore, systemic inflammatory responses mediate the feeding-aversive behavioral effects through this circuit, which are in part caused by changes in the permeability of the blood–brain barrier (BBB). For example, lipopolysaccharide has been demonstrated to cause the release of pituitary adenylate cyclase-activating polypeptide (PACAP), which acts on ADCYAP-1 receptors in the AP and NTS to induce nausea and vomiting [26]. Corticosteroids, in addition to reducing systemic inflammation, are also thought to benefit the symptoms of nausea by blocking the biosynthesis of prostaglandins, which when elevated in the serum, have been associated with nausea in both humans and animal models [27,28,29,30]. Reducing inflammation in the CNS can influence central emetic circuits, and this may explain why treatments aimed at reducing inflammation provide symptomatic relief for autoimmune attacks on the AP.

### 3.2. Incretins and Gastric Hormones

Glucagon-like-peptide-1 receptor (GLP-1R) agonists have become a first-line option for management of type 2 diabetes mellitus, and are now widely used in the management of obesity [31]. Additionally, these therapies may have neuroprotective effects in the setting of Parkinson’s disease, as shown in both animal models and randomized clinical trials [32,33,34,35,36,37]. The most common reported side-effects of GLP-1 use are nausea and vomiting, which can be explained by both transcriptomic and lesional studies in mice [34,38,39,40,41,42,43,44,45,46]. GLP-1R is expressed on both the GABA-ergic and glutamatergic neuron populations in the AP, which together regulate the induction of satiety and sensation of visceral illness [15,22,34,38,39,40,41,42,43,44,45,46]. Because GLP-1 acts directly on the AP, its associated side-effects include brainstem-localized nausea and vomiting. Consequently, its emetogenic effects might also be ablated by the centrally-acting antiemetics that we suggest for treating CNS lesional and intractable nausea and vomiting. The GLP-1 pathway may also be a future target for therapeutic development in the management of nausea and vomiting.

Similar to GLP-1, gastric peptides like gastric inhibitory peptide (GIP) have a robust role in central nausea and vomiting [Table A1]. These peptides can exert profound effects due to their ability to cross the BBB and interact with their receptors in the NTS [47]. Likewise, GIP, neuropeptide Y (NPY), peptide YY (PYY), and ghrelin exert effects on feeding behaviors through action at the AP [15,22,48]. It is likely that gastric signaling peptides exert significant behavioral effects in part through the action of these peptides on brainstem emetic circuitry, which presents an opportunity for related future study on therapeutic targets. Additionally, there are other peptide hormones that can facilitate emetic signaling through action at the AP and NTS. In murine models, oxytocin exerts anorectic effects when delivered into the fourth ventricle by increasing the gastric pressure via vagal preganglionic neurons [49]. Further examinations of peptides that can cross the permeable BBB at the AP may reveal other mediators of satiety, nausea, and vomiting.

### 3.3. Migraines and CGRP

Calcitonin gene-related peptide (CGRP) is a peptide neurotransmitter expressed throughout the peripheral and central nervous systems that mediates both the nociceptive and viscerosensitive pathways [50]. CGRP has recently been investigated for its role in the pathogenesis of migraine headaches, with recent trials demonstrating strong efficacy in the use of anti-CGRP monoclonal antibodies for migraine prevention [51,52]. The functional CGRP receptor is a complex composed of both calcitonin-like receptor (CLR1) and receptor activity-modifying protein (RAMP1). RAMP1 is responsible for transporting CLR1 to the plasma membrane, and is necessary for its Gs*α*-mediated signal transduction [50]. Nausea and vomiting are commonly associated with migraine headaches, and both CLR1 and RAMP1 are expressed in the AP [53]. Experiments in transgenic mouse models have shown that GDNF family receptor alpha-like (GFRAL)-positive cells in the AP project to CGRP+ cells in the parabrachial nucleus (PBN) [15,54]. This AP-PBN circuit controls taste-aversion behaviors, and is activated in the setting of systemic infection and illness [15,54]. GFRAL receptors exist solely in the NTS and AP, and are stimulated largely through growth differentiation factor 15 (GDF-15), which is released in response to a host of conditions including tumors, chemotherapy, and systemic inflammation [15,55]. When assembled, CGRP has potent effects on central emetic circuitry by acting on CLR1 receptors in the AP, whereas other molecules like GDF-15 also bind to the AP but exert their effects through communication with additional brainstem centers.

### 3.4. Endocannabinoid System

Cannabinoids, particularly Δ9-tetrahydrocannabinol (THC), exert antiemetic effects through activation of cannabinoid type 1 receptors (CB1) most densely located in the medial subnucleus of the NTS and the DMX, but also to a lesser degree in the AP [56]. CB1 is a retrograde neurotransmitter that attenuates the serotonergic response centrally, thereby reducing nausea [57]. While clinicians have attempted to exploit their antiemetic properties in the setting of chemotherapy-induced nausea and vomiting, high doses of cannabinoids can paradoxically produce cannabis hyperemesis syndrome (CHS), a cyclic vomiting syndrome typically managed with first-line antiemetics and THC abstinence [58,59]. The pathophysiology of CHS is unclear, and is thought to be caused by downregulation of both enteric and central endocannabinoid receptors that when present, would normally inhibit the emetic response [60].

### 3.5. Capsaicin and TRPV1

Interestingly, a common feature in the history of patients presenting with CHS is that they find relief by taking very hot showers [61,62]. Transient receptor potential vanilloid-1 (TRPV1) is expressed throughout the peripheral nervous system, including in the gustatory receptors of the tongue [63,64,65]. It is thought to trigger nociception in response to heat (>42.7 °C), changes in pH, and “spicy” chemicals such as capsaicin [63,64,65]. TRPV1 is also expressed in the NTS and AP, and its activation is thought to deplete levels of substance P, a key mediator of emesis through potentiation of NK-1 receptors [66,67]. To this end, topical capsaicin has been successfully used to treat CHS in the Emergency Department, and remains a viable second-line option [68]. Interestingly, alcohol aromatherapy has also shown benefit in reducing nausea. A randomized controlled trial performed in the emergency room setting showed that inhaled isopropyl alcohol improved nausea symptoms and reduced the length of stay when compared to oral ondansetron alone, and appears to have a synergistic effect when combined with ondansetron [69]. The mechanism for this is unclear, but may be due in part to stimulation of TRPV1 receptors through a mechanism similar to that of capsaicin.

## 4. Treatment of Central Nausea and Vomiting

The management of intractable nausea and vomiting, the etiology of which can be localized to the brainstem, starts with providing supportive care, including rehydration and correcting electrolyte/nutritional abnormalities. Monotherapy using oral medications with anti-5HT-3 or anti-D2/D3 activity such as ondansetron, prochlorperazine, promethazine, or metoclopramide should be used as first-line antiemetics. Refractory nausea and vomiting can first be managed by trialing the combination of anti-5HT-3 and anti-dopaminergic medications. The addition of olanzapine as a daily medication is another consideration for patients whose nausea and vomiting is refractory to monotherapy.

Patients who have intractable nausea despite the use of monotherapy and combinations of the widely used antiemetics mentioned above represent a significant therapeutic challenge. The use of longer-acting antiemetics that target 5HT-3 and NK-1, such as palonosetron and netupitant, is considered off-label therapy in patients who are not cancer patients on chemotherapy. These medications subsequently have not been tested outside of the CINV indications, and their prolonged weekly use has also not been studied prospectively. Carefully evaluating medication interactions, unique patient-related factors that could impact drug safety, and financial toxicity are important when considering off-label therapies. Potential impacts on the QT interval and other cardiac impacts of these drugs may need particular attention and screening depending on the patient’s medical history.

**Figure 2:**
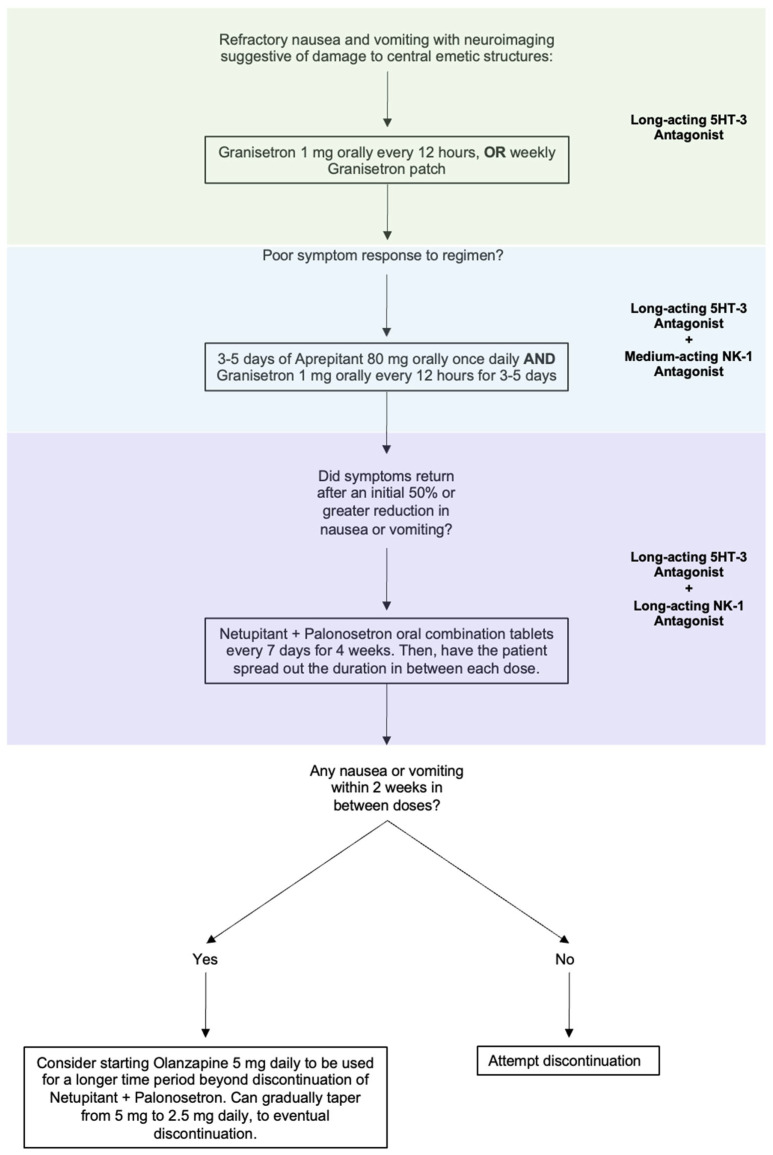
Proposed step-wise treatment algorithm for refractory nausea and vomiting caused by a lesion to CNS vomiting centers.

Intractable nausea and vomiting due to a CNS lesion or injury can be approached by combining 5-HT3 blockers with an NK-1 receptor antagonist. As most patients will have tried and failed ondansetron, likely in combination with D2 blockers, longer-acting medications reserved for use in CINV would be the next agents to trial. Granisetron orally every 12 h is a reasonable first step to see if a longer acting 5HT-3 antagonist improves the nausea and vomiting. The granisetron patch is also available as a weekly therapy, which may be an option in the right clinical circumstances. If long-acting 5HT-3 antagonism is unsuccessful, moving to combined therapy would be the next step. Given the off-label nature, cost, and unknown safety profile of the following medications in combinations outside of their indicated uses, we recommend a sequential therapeutic trial to target the symptoms that the patient is experiencing (Figure 2).

One important consideration when prescribing netupitant and palonosetron combination tablets is the patient’s access to care. One advantage relevant to medication adherence is that the tablets need only be taken weekly. Although the cost of the combination tablets is more expensive than the generically available options, it has been shown that overall they have a net cost-saving effect [70]. This is owed to their improved efficacy in treating CINV, which prevents other costs that are typically associated with recurrence of symptoms while on standard treatment. However, clinicians should remain cognizant of the limited availability of combined tablets, and that some forms of insurance may require demonstration of the existence of refractory symptoms despite having received the standard-of-care.

## 5. Conclusions

The area postrema serves as a critical hub in the central regulation of nausea and vomiting, integrating signals from the serum, gastrointestinal tract, and multiple CNS circuits. Central nausea and vomiting syndromes, such as those that occur following ischemia, mass effect, or demyelination affecting the AP, result in profound dysfunction of emetic regulation. This direct damage to central emetic machinery creates a syndrome of intractable nausea and vomiting resistant to first- and second-line antiemetic medications. However, our cases suggest that combined HT-3 and NK-1 blockade, a regimen effective for CINV, can also manage area postrema syndrome. The mechanism through which this is achieved is likely a combination of the inhibition of central pro-emetic neurons with the prevention of pro-emetic feedback from enteric neurons.

As knowledge of the AP’s structure grows, new targets may prove to be equally effective in treating centrally mediated hyperemesis. Emerging evidence from molecular studies has revealed potential therapeutic targets such as the gastric inhibitory polypeptide receptor (GIPR), transient receptor potential vanilloid-1 (TRPV1), and glucagon-like peptide-1 (GLP-1) receptors. These receptors are all expressed in the AP and can independently modulate nausea and vomiting by acting as chemical triggers. Further exploration into the clinical application of these targets may provide broader therapeutic options for patients with central nausea and vomiting syndromes that are refractory to first- and second-line medications.

Important limitations of this review include the small number of case series, which is in part due to the rarity of APS stemming from vascular or neoplastic insults. The lack of randomized data makes it difficult to determine whether using the combination of palonosetron–netupitant is superior to the standard-of-care at a wider scale. Similarly, the small sample size may mask the side-effect profiles of using these medications outside of CINV. Although 5HT-3 antagonists like ondansetron and dolasetron are known to cause QT-interval prolongation, this has not been demonstrated with palonosetron [71,72]. However, there remains a theoretical risk of serotonin syndrome. NK-1 receptor antagonists, including netupitant, are known inhibitors of the cytochrome P450 3A4 (CYP3A4) enzyme, a major player in drug metabolism [73,74]. Notably, the dosage of glucocorticoids is adjusted when given in conjunction with NK-1 antagonists, and so this metabolic effect should be considered, depending on the other medications in the patient’s regimen [73].

Intractable nausea and vomiting present a challenging management scenario for clinicians, as direct damage to the area postrema or other portions of the dorsal vagal complex creates an environment that responds poorly to antiemetics. In general, when refractory nausea and vomiting occurs due to central or peripheral triggers, most guidelines and reviews recommend using long-acting 5HT-3 antagonists [75,76,77,78,79,80]. Additionally, non-serotoninergic options may also be added to broaden the spectrum of antiemetic action; these may include benzodiazepines, cannabinoids, antihistamines, or dopaminergic medications [75,76,77,78,79,80]. Leveraging the contemporary understanding of central nausea and vomiting circuits may help provide relief for patients with direct AP injury whose symptoms initially fail to resolve with first-line medications.

## Data Availability

No new data were created or analyzed in this study. Data sharing is not applicable to this article.

## Appendix A

**Table A1:** Receptors expressed in peripheral and central neural circuits implicated in mediating the sensation of nausea and execution of the vomiting response.

Receptor	Distribution	Mechanism	Treatment Implications	References
Adenylate Cyclase Activating Polypeptide Receptor (ADCYAP-1-R)	Central: AP/NTS, vestibular nucleus.	ADCYAP-1R is a G-protein coupled receptor that responds to PACAP, a neuropeptide involved in the stress response and trigeminovascular system. Activation of the ADCYAP-1-Rs in the AP causes activation of signaling pathways in the AP/NTS involved in the emetic response. PACAP is released in response to LPS, with the downstream effect of modulating aversive feeding behaviors.	Antagonism could be a future potential target. There are very early in vitro and pre-clinical studies using small-molecule antagonists.	[26]
Aquaporin-4 (AQP-4-R)	Central: Astrocytes and ependymal cells, particularly at the end feet processes surrounding CVOs—there is a very high density within the AP. Also found in the NTS and hypothalamus.	Gate water transport in the CNS and are critical to the integrity of the BBB. Regulate the fine osmotic balance within the CNS. In NMOSD, severe edema results from antibodies directed at AQP-4, leading to intractable nausea and vomiting if the lesion is near the AP.	Could be a potential future target for selectively limiting brain edema following CNS injury and thus limit some effects from the disruption in BBB integrity.	[19,81]
Calcitonin Gene-Related Peptide (CGRP-R)	Peripheral: Enteric nervous system, vagal afferents, trigeminal nerve, dorsal root ganglia. Central: Parabrachial nucleus (PBN), NTS, AP.	CGRP is released by trigeminal and vagal afferents, playing a key role in the nausea induced by migraines. CGRP-containing neurons in PBN are activated by noxious stimuli and project to the NTS via glutamatergic synapses. Circulating gastric hormones can cause the release of CGRP in the AP, which stimulates the PBN. The PBN sends projections to subcortical areas that process emetic and interoceptive signals. This leads to food aversion behaviors in animal studies	CRGP antagonists are commonly used for migraine treatment and have a well-established role in central pain sensitization. The prominent central action may indicate a potentially useful adjuvant for central causes of N/V.	[15,50]
Cannabinoid-1 (CB1-R)	Central: AP, NTS, DMX, hypothalamus, hippocampus. Peripheral: Enteric nervous system, GI epithelial cells.	CB1 is a retrograde neurotransmitter system that acts to limit serotonin release centrally, thus attenuating nausea and vomiting through suppression specifically within the NTS. Administration of THC directly into the brainstem mitigates CINV in ferrets through activation of CB-1-Rs. Some, albeit weak, evidence to suggest downregulation of CB1 leads to cannabis hyperemesis syndrome. Alternative hypotheses include desensitization with repeated exposure. In general, receptor activation stimulates feeding behavior through interactions between the NTS and PBN.	Used in CINV, especially in refractory situations. Although there are complex central interactions, CB1-Rs are a potential target for antiemetic effects.	[16,56,57,60,82,83]
Dopamine-2 (D2-R)	Central: Distributed widely in the AP, NTS, DMX. Peripheral: Vagal afferents, GI tract. Also located throughout the cortex, and may play a role in signaling from the DVC to higher-order structures, such as the insula.	Receptors in the AP are activated by circulating emetic stimuli, leading to intracellular signaling cascades and activation of the vomiting reflex. Receptors in the DMX are involved in modulating gastric tone and phasic contractions.	Demonstrated efficacy in certain central causes of nausea and vomiting, such as migraines. Current anti-dopaminergic agents act centrally (contributing to extrapyramidal side-effects) but also exert some 5HT-3 blocking effect.	[3,14,82,84]
GDNF Family Receptor-Alpha-Like (GFRAL)	Central: AP, NTS, PBN.	Activated by GDF-15, a stress response cytokine, the production of which is stimulated by metformin, systemic inflammation, various tumors, and chemotherapy. Stimulation reduces feeding behavior and can induce nausea and vomiting. GDF-15 has wide-ranging effects throughout the body, but activation of GFRAL is specific to AP/NTS.	GDF-15 monoclonal antibody-abated cisplatin induced nausea and vomiting in non-human primate model. Also thought to play a role during pregnancy-related nausea and vomiting. GFRAL antibody has been used in mouse model to attenuate tumor-associated cachexia.	[15,22,55,85]
Ghrelin-R	Central: AP, NTS, hypothalamus. Peripheral: Vagus nerve, GI mucosal cells.	Stimulates receptors directly within the AP to activate feeding behaviors in animal models. Also modulates AP/NTS activity through stimulation by vagal afferents. Requires a functional AP to mediate effects, as seen in lesional studies. Peripherally it has prokinetic effects.	Agonists are currently being investigated for both prokinetic and anti-nausea uses.	[1,48]
GLP-1-R	Central: Mainly in the AP and NTS, but can also be found in PBN. Peripheral: Vagal afferents.	GLP-1 is released by gut enterochromaffin cells after meals and exerts some effect locally to slow GI transit. However, it also mediates effects through glutamatergic projections in the AP/NTS, leading to appetite suppression. Weight-loss effect of agonists is mediated through the AP/NTS, as central lesions in animal models ablate the effects of these peptides. Activation mediates avoidance of aversive substances in mouse models, likely through action in the PBN.	Example of how profound the central action of gastric peptides can be. Avoiding use of these agonists with respect to any central cause of nausea and vomiting is reasonable. Antagonism may be a potential target for future therapeutics.	[15,45,47]
Glucose-Dependent Insulinotropic Polypeptide (GIP-R)	Central: AP, NTS. Peripheral: GI vagal afferents.	Like many gastric peptides, GIP readily crosses the BBB to exert effects at the AP. Agonism causes activation of GABA-ergic neurons in the AP/NTS to inhibit emetic pathways in animal models of CINV. Further studies show GIP agonism does not diminish the weight-loss effects of anorectic peptides such as GLP-1 agonists.	Additional example of a centrally acting gastric hormone that attenuates nausea, with some pre-clinical utility in CINV models. When administered with the anorectic peptides being used for weight loss, reduces nausea and vomiting without affecting hypophagia.	[22,47,86]
Histamine Receptor type 1 (H1)	Central: AP, NTS, DMV, vestibular nuclei. Peripheral: Vagal afferents.	H1-R mediated depolarization of neurons within the AP/NTS increases glutamatergic signaling, which leads to vestibular-induced nausea and vomiting. Glutamatergic signaling within the NTS is highly involved in vestibulo-autonomic reflex during vestibular stimulation. Activation of vagal afferents in the GI tract relays signals that induce the emetic response via the AP/NTS. In animal studies, intraventricular administration of histamine caused vomiting that was prevented with either AP ablation or anti-histamine pretreatment.	Many antiemetic agents have broad effects blocking both central H1 and M1 receptors in the vestibular circuit (i.e., prochlorperazine, diphenhydramine).	[3,14,33,82,84]
Interleukin-6 (IL-6-R)	Central: AP, hypothalamus, organum vasculosum of the lamina terminalis.	Activation of IL-6 receptors, particularly in the AP, stimulates excitatory signaling within the NTS to activate the emetic pathway. Stimulation in pre-clinical models induces cachexia. In mouse tumor models, IL-6R-blocking antibodies ameliorated anorexic effects and prolonged lifespan. Partial mechanistic explanation for how systemic inflammation can mediate nausea, vomiting, and centrally mediated food avoidance.	This is one of the potential pathways by which steroids can reduce central nausea, as they limit the release of cytokines onto CVOs.	[1,24]
Mu Opioid Receptor	Central: AP, NTS. Peripheral: Vagal afferents, GI tract.	Stimulation of the mu opioid receptor causes vomiting in animal models that is reduced with AP ablation but not with vagotomy. Three mechanisms: (1) Direct stimulation of DVC. (2) Slowing of GI transit. (3) Stimulation of the vestibular apparatus.	Demonstrates the importance of avoiding opioids in central nausea and vomiting.	[3]
Muscarinic Acetylcholine Receptor Type 1 (M1)	Central: AP, NTS, vestibular nuclei. Peripheral: Vagal afferents in the GI tract.	Cholinergic signaling induced by vestibular stimuli leads to excitatory signaling within the AP/NTS. In feline studies, intraventricular administration of M1 agonist caused emesis, and this was prevented with ablation of AP.	Scopolamine is commonly used in motion-sickness and post-operative nausea, acting centrally via suppression of vestibular signaling.	[3,14,82,84]
Neurokinin-1 (NK-1-R)	Central: AP, NTS, DMX. Peripheral: myenteric plexus in the enteric nervous system, dorsal root ganglia, sympathetic ganglia.	Substance P is released from enterochromaffin cells in the gut, although to a far lesser extent than 5HT-3. Substance P release activates NK-1R’s in the AP/NTS, stimulating emetogenic reflex. Predominantly central action in CINV, as evidenced by ferret studies showing no response to NK-1R antagonists when the ability to cross the BBB is removed.	Widely accepted as effective for treatment of the delayed phase of CINV. Also relevant in gastroparesis, with no impact on GI motility, indicating prominent centrally acting effects. Also thought to be involved in the complex and poorly understood cyclic vomiting syndrome. NK-1R antagonists given during the prodromal phase significantly reduce the duration of vomiting episodes.	[2,3,11,82,87]
Neuropeptide-Y-2 (NPY-2-R)	Central: AP and NTS. Peripheral: Vagal afferents.	Peptide YY (PYY) is secreted from GI neuroendocrine cells. NPY2-R, stimulated by three ligands: PYY, NPY, and pancreatic polyprotein process. PYY crosses the BBB, and then exerts effects on receptors in the AP, predominantly to evoke anorectic effects in animal models. These effects cease with AP ablation. Also involved in the cardiovagal response, leading to vasoconstriction and hypotension.	Further evidence of the strong connection between gut hormones and the AP. Potential future target.	[3,88]
Oxytocin-R	Central: AP, NTS, insula. Peripheral: Vagal afferents in the GI tract.	Stimulation has anorectic effects through increasing gastric pressure via vagal preganglionic signaling in the DVC and vagal afferents. There is evidence that activation can alter behavior through insular signaling pathways (food intake, etc.).	There are very early pre-clinical studies looking at oxytocin-R antagonists for modulating nausea and visceral pain sensations.	[5,49]
Prostaglandin E2-R	Mainly found peripherally in the GI tract and vagal afferents. Central: AP, NTS.	Prostaglandin E2 (PGE2) is derived from cell membrane phospholipids in response to inflammation, especially in the GI tract. Circulating PGE2 stimulates receptors in the AP and leads to activation of nausea and vomiting pathways. PGE2 levels correlate with nausea and vomiting during pregnancy.	One of the many mechanisms by which glucocorticoids exert antiemetic effects: by reducing formation of prostaglandins during inflammation.	[3,82]
Serotonin Type 3 (5HT-3-R)	Peripheral: High receptor concentration on vagal afferents. Central: Receptors also located centrally, to a lesser extent, in the NTS, AP, and DMX.	5HT-3 is mainly released from enterochromaffin cells in the GI tract. Initial cascade relays visceral sensation to the area postrema (AP) and NTS through vagal afferents which modulate glutamatergic signaling within the NTS. 5HT-3-R is an ion channel that causes depolarization and the release of multiple neurotransmitters such as Substance P and cholecystokinin onto the NTS.	5HT-3 antagonists are one of the mainstays of treatment of CINV and nausea and vomiting in general. Dexamethasone has been shown to modulate vagal 5HT-3 activity.	[2,3,48,89,90]
Serotonin Type 4 (5-HT4-R)	Peripheral: Mainly within the GI tract (intestinal epithelial cells and enteric neurons). Central: AP, NTS, DMX.	Activation of the receptor enhances acetylcholine release and stimulates GI motility, overall prokinetic effects. Central receptors modulate the sensory input from the vagus nerve, reducing nausea.	Many medications available have either full or partial agonist effects. Useful in gastroparesis, irritable bowel syndrome, and postoperative ileus.	[84]
Toll-like Receptor 4 (TLR-4)	Central: Found in CVOs, notably the AP.	Activated by inflammatory mediators such as LPS, increasing permeability of CVOs and neuroinflammation. Thought to play a role in central parasitic infections (trypanosomiasis).	Additional mechanism by which steroids act to reduce nausea and vomiting in the setting of inflammation.	[25]
Transient Receptor Potential Vanilloid-1 (TRPV-1)	Central: AP, NTS Peripheral: Presynaptic myelinated and unmyelinated vagal afferents, dorsal root ganglia, trigeminal ganglia.	Activated by capsaicin, heat, and low pH. Activation leads to release of substance P onto NK-1 R’s, both peripherally and in the AP/NTS. Also causes the release of GCRP. Overstimulation leads to depletion of substance P and both peripheral and central internalization of the NK-1 Rs, providing an antiemetic effect. Central TRPV-1 receptors are also thought to be partially stimulated by cannabinoids, which may explain the antiemetic effects of cannabis.	Capsaicin cream is used in cannabis hyperemesis syndrome. The mechanism is somewhat unclear, although it likely involves peripheral desensitization of TRPV-1 through depletion of substance P. Potential adjuvant to modulate central NK-1 R activity through peripheral depletion of substance P.	[57,65,83,91,92]
